# Assessment of Urinary Deoxynivalenol Biomarkers in UK Children and Adolescents

**DOI:** 10.3390/toxins10020050

**Published:** 2018-01-23

**Authors:** Maria Papageorgiou, Liz Wells, Courtney Williams, Kay White, Barbara De Santis, Yunru Liu, Francesca Debegnach, Brunella Miano, Giorgio Moretti, Stephanie Greetham, Carlo Brera, Stephen L Atkin, Laura J Hardie, Thozhukat Sathyapalan

**Affiliations:** 1 Department of Diabetes, Endocrinology and Metabolism, University of Hull, Brocklehurst Building, Hull Royal Infirmary, Anlaby Road, Hull HU3 2RW, UK; Liz.Wells@hull.ac.uk (L.W.); Thozhukat.Sathyapalan@hyms.ac.uk (T.S.); 2 Division of Epidemiology and Biostatistics, LICAMM, School of Medicine, University of Leeds, Leeds LS2 9JT, UK; Courtney.Williams18@outlook.com (C.W.); K.L.M.White@leeds.ac.uk (K.W.); L.J.Hardie@leeds.ac.uk (L.J.H.); 3 Department of Food Safety, Nutrition and Veterinary Public Health, Food Chemical Risk, Istituto Superiore di Sanità, Viale Regina Elena, 299, 00161 Rome, Italy; Barbara.Desantis@iss.it (B.D.S.); Francesca.Debegnach@iss.it (F.D.); Carlo.Brera@iss.it (C.B.); 4 Department of Environmental Medicine, Hainan Medical University, 3 Xueyuan Road, Haikou 571199, China; Liuyunru@126.com; 5 Public Health and Risk Analysis, Istituto Zooprofilattico Sperimentale delle Venezie, Viale dell’Università, 10, 35020 Legnaro, Italy; Bmiano@izsvenezie.it; 6 Istituto Zooprofilattico Sperimentale dell’Umbria e delle Marche, Via G. Salvemini 1, 06126 Perugia, Italy; G.Moretti@izsum.it; 7 Echuca Regional Health, Service Street, Echuca 3564, Australia; Sgreetham@erh.org.au; 8 Weill Cornell Medicine in Qatar, Education City, P.O. Box 24144, Qatar; Sla2002@qatar-med.cornell.edu

**Keywords:** mycotoxins, deoxynivalenol, *Fusarium graminearum*, children, adolescents, biomonitoring, This study showed moderate mean levels of DON in urine samples of children aged 3–9 years and adolescents aged 10–17 residing in an urban area of the UK. However, the high frequency of DON detection, together with the high maximum urinary biomarker concentrations and estimated dietary exposure of DON are concerning for some young individuals within this cohort.

## Abstract

Deoxynivalenol (DON), the mycotoxin produced mainly by *Fusarium graminearum* and found in contaminated cereal-based foodstuff, has been consistently detected in body fluids in adults. Available data in children and adolescents are scarce. This study assessed urinary DON concentrations in children aged 3–9 years (*n* = 40) and adolescents aged 10–17 years (*n* = 39) in the UK. Morning urine samples were collected over two consecutive days and analysed for free DON (un-metabolised form), DON-glucuronides (DON-GlcA), deepoxy deoxynivalenol (DOM-1), and total DON (sum of free DON, DON-GlcA, and DOM-1). Total DON was detected in the urine of >95% of children and adolescents on both days. Mean total DON concentrations (ng/mg creatinine) were 41.6 and 21.0 for children and adolescents, respectively. The greatest total DON levels were obtained in female children on both days (214 and 219 ng/mg creatinine on days 1 and 2, respectively). Free DON and DON-GlcA were detected in most urine specimens, whereas DOM-1 was not present in any sample. Estimation of dietary DON exposure suggested that 33–63% of children and 5–46% of adolescents exceeded current guidance regarding the maximum provisional tolerable daily intake (PMTDI) for DON. Although moderate mean urinary DON concentrations were shown, the high detection frequency of urinary DON, the maximum biomarker concentrations, and estimated dietary DON exposure are concerning.

## 1. Introduction

Deoxynivalenol (DON) belongs to a large group of mycotoxins named trichothecenes, which represent the main group of Fusarium toxins. Humans are exposed to DON predominantly by consuming DON-contaminated cereal grains (i.e., maize, wheat, barley, and oats) and cereal-based processed food (e.g., porridge, cereals, bread, and malt beer) [1]. Animal studies have shown that the main adverse effects of DON exposure include vomiting, nausea, anorexia, delayed growth, neurological changes, and impairments in immune and reproductive function [2], although it is uncertain whether humans experience the same effects. Given these potential associated health risks and high frequency of DON contamination worldwide [3,4], DON exposure is regarded as an important food safety issue. To protect consumers’ health, the Scientific Committee on Food (SCF) set the tolerable daily intake (TDI) of DON at 1 μg/kg body weight (b.w.)/day in December 1999 [5]. The Joint Food and Agriculture Organization of the United Nations (FAO)/World Health Organisation (WHO) Expert Committee on Food Additives (JECFA) evaluated DON during the 72nd meeting in 2010 and converted the provisional maximum tolerable daily intake (PMTDI) of DON to a group PMTDI of 1 μg/kg body weight (b.w.)/day for DON and its metabolites 3-Acetyldeoxynivalenol (3-Ac-DON) and 15-Acetyldeoxynivalenol (15-Ac-DON), and emphasised the importance to investigate DON exposure in different countries and populations [6]. 

The assessment of DON exposure traditionally included data on DON contamination levels in food combined with population data on food consumption [7,8]. This approach requires available food contamination data, omits exposure routes other than dietary intake (e.g., inhalation of contaminated air and skin contact with contaminated surfaces) [3], and assumes the homogeneous distribution of mycotoxin within any given food item [9,10]. The measurement of DON-related biomarkers in biological fluids using analytical methods with high sensitivity has advanced the assessment of DON exposure in humans [11,12]. DON is detectable in serum in high amounts immediately after ingestion, but it is rapidly cleared from the bloodstream [13]. The main fraction of DON is excreted in the urine. In humans, DON is present in its un-metabolised form or “free” DON and as glucuronide conjugates (DON-GlcA) including deoxynivalenol-3-glucuronide (DON-3-GlcA) and deoxynivalenol-15-glucuronide (DON-15-GlcA). De-epoxy DON (DOM-1), the detoxification product of DON formed by gut microbiota in animals and humans, can also be measured in urine; although it has not been consistently detected in human studies [14,15,16,17]. The discrepant results in human studies may be due to differences in analytical procedures and their sensitivity or may reflect the presence or absence of gut microbiota necessary for DON detoxification through this pathway in different populations [15,17]. Further research is required to explore the occurrence of DOM-1 in relevant population groups.

An increasing number of studies which assessed urinary DON levels and its metabolites in adult populations showed exposure levels close to, or even higher than, the PMTDI [12,15,18]. There is also interest in investigating DON exposure in potentially susceptible population groups due to their physiological status (e.g., pregnancy) [19]; dietary habits (e.g., vegetarians) [18]; and age (i.e., children and adolescents) [20,21,22]. Children are considered to be at risk for greater relative DON exposure than adults due to their higher food and fluid intake per kg of body weight compared to adults, child/adult metabolic differences, limited ability for detoxification, and on-going bodily system development including the immune and central nervous system [23]. Currently, data on DON exposure among children and adolescents are limited [21,22], despite its potential effects on disease development over the lifecourse [2].

This study aimed to analyse and compare levels of DON and its metabolites in the urine of children aged 3–9 years and adolescents aged 10–17 years in the UK. A secondary goal was to investigate the relationship between demographic and anthropometric characteristics, as well as dietary factors and DON concentrations in these young age groups. 

## 2. Results

### 2.1. Baseline Characteristics

A total of 50% of the 40 children aged 3–9 years and 49% of the 39 adolescents aged 10–17 years were males. Between female and male children, there were no differences in height (female children: 115 ± 3 cm, male children: 121 ± 4 cm; *p* = 0.27); weight (female children: 22 ± 2 kg, male children: 24 ± 2.0 kg; *p* = 0.53); or BMI (female children: 16.1 ± 0.5 kg/m^2^, male children: 15.8 ± 0.5 kg/m^2^; *p* = 0.69). In contrast, the mean height, weight, and BMI for adolescent females (height: 160 ± 2 cm; weight: 57 ± 2 kg and BMI: 22.0 ± 0.9 kg/m^2^) were significantly higher than the mean values of these variables in adolescent males (height: 155 ± 4 cm; weight: 46 ± 3 kg and BMI 18.5 ± 0.6 kg/m^2^; all *p*-values from 0.003 to 0.050), which is in line with the sex differences seen during adolescence. All variables were significantly higher in adolescents than in children for both sexes (*p*-values between <0.0001 and 0.002), confirming the distinct anthropometric characteristics of these age groups. In children aged 3–9 years old, 5%, 52.5%, and 42.5% reported light, moderate, and high physical activity levels, respectively, whereas the respective percentages for adolescents 10–17 years were 20.5%, 38.5%, and 41%. 

### 2.2. DON Biomarker Levels in Urine Samples 

**Figure 1:**
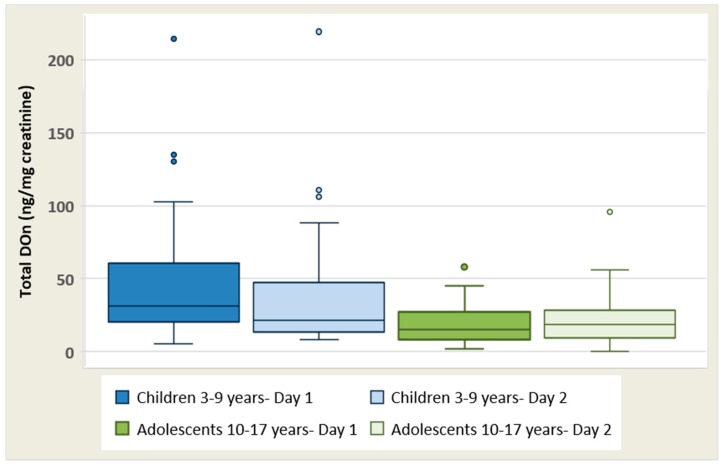
Box plot of creatinine-adjusted total DON concentrations in children aged 3–9 years for day 1 (dark blue) and day 2 (light blue) and adolescents aged 10–17 years for day 1 (dark green) and day 2 (light green). The band inside the box is the second quartile (P50, median). Dots are suspected outliers. Whiskers are set from minimum to maximum value. The bottom and the top of the box are the first and third quartiles (P25 and P75).

Non-adjusted and creatinine-adjusted mean urinary DON levels on day 1 and 2 in children aged 3–9 years (*n* = 40) and adolescents aged 10–17 years (*n* = 39) are presented in Table 1. Total DON was identified in 100% of children aged 3–9 years (*n* = 40) on days 1 and 2 and 100% of adolescents aged 10–17 years on day 1. On day 2, total DON was identified in 95% of females (19 out 20 females) and 100% of males (*n* = 19). Overall, creatinine-adjusted mean levels of total DON (ng/mg creat) for both days (mean values for day 1 and 2) were 41.6 for children aged 3–9 years (*n* = 40) and 21.0 adolescents 10–17 years (*n* = 39); with children aged 3–9 years presenting with significantly higher levels than those reported in adolescents 10–17 years (*p* = 0.001). The considerable differences between the 50th (median) and the 75th percentiles indicate a relatively wide range of exposure across the groups (Figure 1). Furthermore, the maximum levels of total DON were extremely high among female children on both days; day 1: 214 total DON (ng/mg creat) and day 2: 219 total DON (ng/mg creat) (Table 1; Figure 1). Overall, individuals who reported to have high total DON concentrations on day 1 also tended to have similarly high total DON concentrations on day 2. Total DON concentrations (ng/mg creat) did not differ between day 1 and 2 in children (females, *p* = 0.15; males, *p* = 0.91) or adolescents (females, *p* = 0.55; males, *p* = 0.21). 

Free DON (>limit of quantification or LOQ 0.25 ng/mL) and DON-GlcA (>LOQ 0.50 ng/mL) were detected in most of the urine specimens, whereas DOM-1 (>LOQ 0.50 ng/mL) was not present in any sample of children or adolescents for day 1 or day 2. DON-GlcA represented 77–88% of the total DON, whereas free DON contributed the remaining 12–23% of total DON levels for both days in the absence of DOM-1. These results confirm that glucuronide conjugation is an important route for DON excretion in children and adolescents consistent with reports in adults [24].

Mean urinary free DON concentrations (ng/mg creat) were 12.4 and 6.8 on day 1 and day 2 in female children and 7.5 and 6.9 for day 1 and 2 in male children, respectively. In adolescents, mean urinary levels of free DON (ng/mg creat) for days 1 and 2 were 2.8 and 2.7 among females and 3.7 and 5.3 among males, respectively. Free DON concentrations were not significantly different between day 1 and day 2 in children (females, *p* = 0.13; males, *p* = 0.85) or adolescents (females, *p* = 0.98; males, *p* = 0.21). Furthermore, mean free DON concentrations for days 1 and 2 were significantly higher in children aged 3–9 years than those reported in adolescents 10–17 years (children: 8.4 ± 9.7 ng free DON/mg creat and adolescents: 3.6 ± 2.8 ng free DON/mg creat; *p* = 0.03) (Table 2). 

Mean urinary DON-GlcA (ng/mg creat) concentrations were 43.9 and 38.2 on days 1 and 2 in female children and 26.5 and 25.2 for days 1 and 2 in male children, respectively, with no differences found between the two days (*p*-values 0.12–0.63). In adolescents, mean urinary levels of DON-GlcA (ng/mg creat) for days 1 and 2 were 16.7 and 17.6 among females and 16.1 and 21.5 among males, respectively. Among this adolescent population, mean DON–GlcA (ng/mg creat) levels were not significantly different between days 1 and 2 (females, *p* = 0.78; males, *p* = 0.20). Furthermore, mean DON-GlcA (ng/mg creat) levels (pooled data for both days) were significantly higher in children than those reported in adolescents (children: 32.2 ± 32.1 ng free DON/mg creat and adolescents: 17.3 ± 11.8 ng free DON/mg creat; *p* = 0.007) (Table 2). 

### 2.3. Estimated Dietary Intake of DON Based on Urinary Analysis 

A risk assessment was performed by comparing the estimated dietary intake of DON with the PMTDI of 1 μg/kg b.w./day (Table 3). The estimated intake for total DON varied considerably depending on the assumptions regarding urine volume for both age groups, with children aged 3–9 years presenting with a higher dietary intake of DON compared to adolescents 10–17 years. For children, the mean estimated dietary intake of DON was 1.0, 1.5, and 2.0 μg/kg·b.w./day, when urine volume (mL/kg b.w./h) was estimated to be 1, 1.5, and 2, respectively [22]. Similarly, for adolescents, the mean estimated dietary intake of DON was 0.5, 0.6, and 1.0 μg/kg·b.w./day, when urine volume (mL/kg b.w./h) was estimated to be 0.5, 0.75, and 1, respectively [25]. Depending on the estimated urine volume used, 33–63% of children and 5–46% of adolescents were estimated to exceed the PMTDI of 1 μg/kg b.w./day.

### 2.4. Correlation between Urinary DON, Demographic and Anthropometric Characteristics

Significant correlations were shown between urinary mean total DON (ng/mg creat) for both days and age (children > adolescents, *p* = 0.001), height (r = −0.307; *p* = 0.006), and weight (r = −0.228; *p* = 0.043), but not sex (r = 0.044; *p* = 0.70). 

### 2.5. Correlation between Urinary Total DON and Food Intake

Table 4 presents the consumption of foods that are commonly contaminated with DON and therefore, these are the main contributors to the dietary DON exposure. Total DON (ng/mg creat) was correlated with pizza intake (r = 0.298, *p* = 0.008) and total dietary intake of food commonly contaminated with DON (r = 0.309, *p* = 0.006). 

## 3. Discussion

Children and adolescents are alleged to be exposed to great amounts of DON due to their high relative consumption of commonly DON-contaminated foodstuff and may be sensitive to DON-associated toxic effects including but not limited to vomiting, nausea, immunosuppression, and delayed growth due to immature biological processes and the incomplete development of bodily systems [2,23]. In this study, although moderate mean urinary DON concentrations were detected in children aged 3–9 years and adolescents aged 10–17 years, the high frequency of DON detection (95–100%) combined with the high maximum biomarker concentrations and estimated dietary DON exposure (based on urinary levels) are of health concern [2]. 

In comparison to the other European sites in the original study [20], children aged 3–9 years (mean: 41.6 ng/mg creat, max: 219.0 ng/mg creat) and adolescents 10–17 years (mean: 21.0 ng/mg creat, max: 95.6 ng/mg creat) in our UK cohort presented with the highest concentrations of total DON concentrations in urine. Specifically, among both Italian (*n* = 40) and Norwegian children (*n* = 40) aged 3–9 years, mean total DON concentration was 14.0 ng/mg creat, with greater maximum levels reported in the Norwegian cohort (76.1 vs. 56.2 ng/mg creat). Mean concentrations of total DON (ng/mg creat) for adolescents 10–17 years were 12.2 (maximum: 75.7 ng/mg creat) in Italy and 7.3 (maximum: 29.4 ng/mg creat) in Norway. In a small paediatric study (*n* = 16) in Spain, Rodriguez-Carrasco et al. [22] reported similar mean and maximum total DON concentrations in urine samples of children aged 8–14 years (mean 27.8 ng/mg creat, maximum: 84.5 ng/mg creat) to those reported in the adolescent group aged 10–17 year old in the present study (mean: 21.0 ng/mg creat, maximum: 95.6 ng/mg creat). In contrast, mean urinary total DON concentrations in our UK cohort are clearly higher than those reported for children aged 3–12 years (*n* = 155) from Belgium (mean: 5.5 ng/mg creat, range: 0.6–27.4 ng/mg creat) [21]. Similar to the results from studies in European paediatric populations, DON has been consistently detected in the urine of young populations in America [26] and Africa [27]. 

In addition to the frequency of detection and total DON concentrations in the urine, by using previously published methods [21,28] and several estimations about urine volume [25], we estimated the dietary intake of DON and compared these results with the PMTDI (1 μg/kg b.w./day) [6]. Unsurprisingly, differences in estimated urine volumes introduced great variability in the estimated intake for DON. Indeed, depending on the estimated urine volume used, 33–63% of children and 5–46% of adolescents were estimated to exceed the PMTDI of 1 μg/kg b.w./day. These results suggest possible health risks for these young UK population groups, especially for children aged 3–9 years. The percentages exceeding the PMTDI for DON in children and adolescents are much higher than previous estimations reported in UK adults. Wells et al. (2017) [18] analysed urine samples of 31 adults in the UK and <3% exceeded the PMTDI. In an earlier study conducted by Turner et al. in the UK, 17% of the participants exceeded the PMTDI (0.008–1.24 μg/kg·b.w./day), although the number of urine samples analysed was small (*n* = 6) [13]. Compared to the estimations of PMTDI in paediatric populations, our results are consistent with those reported in children of 3–12 years in a Belgian cohort (56–69% exceeded PMTDI, 0.11 and 19.57 μg/kg·b.w./day) [21], but higher than those reported in Spanish children aged 8–14 years (*n* = 16) (0.06 and 1.07 μg/kg·b.w./day, 22% exceeded PMTDI) [22]. It is important to note that estimations of dietary intake of DON should be interpreted after considering the limitations of the available approaches [28,29]. For example, our calculations were based on a 72% excretion rate of DON, previously shown in a UK-based population [13], and did not account for inter-individual variations. We clearly demonstrated that assumptions for urine volumes introduce variability, making the comparison of the results challenging. Furthermore, true urine excretion volumes of our participants may deviate to some extent from the estimated urine volumes tested for this analysis.

Overall, the mixed results of these biomonitoring studies may be partially explained by differences in dietary habits of the population residing in different countries or even different regions within a country, but also seasonal variations in dietary intake within a given population [16,30]. Different contamination of foodstuff with DON due to factors such as climate conditions, and cultivating and processing practices are also possible reasons for the inconsistent results reported in the literature [27,31,32]. However, we cannot provide a more direct comparison due to differences in study demographics (e.g., age ranges), analytical methods, and quantification limits. Future large-scale studies in children and adolescents from different parts of the same country and during different seasons throughout the year are needed to produce representative and generalizable data on DON exposure within a country and allow for data comparison between countries. 

Our study provides useful insight into DON and its metabolites in urine. DON-GlcA was detected in most urine specimens during both data collection days and contributed between 77% and 88% to the total DON levels, confirming that glucuronides are the main DON metabolites. These results are consistent with those from studies in UK adults [18,24] and paediatric populations in other countries [21]. For example, in our earlier study, DON-GlcA made a significant contribution to the total DON concentration (83–84%) in adults aged 18–64 years (*n* = 31) [18]. Previous work has detected DON glucuronides at different positions, with DON-GlcA in positions 3 and 5 (DON-3-GlcA and DON-15-GlcA) being the most commonly detected forms [21,29]. In the present investigation, DON-GlcA concentrations were determined as the difference between total DON and free DON values for each individual (indirect method), and as such, information for DON glucuronides at different positions is unavailable. 

In this study, DOM-1 was not detected in children aged 3–9 years or adolescents aged 10–17 years, suggesting that DOM-1 is not a major route of detoxification in our UK-based paediatric population. In agreement with these findings, DOM-1 was not present in urine samples of children from Italy [20] or urine samples of UK adults [11,24]. In contrast, DOM-1 was detected in 34% of French farm workers [15], 38–60% of German mil workers and controls [17], and 27% of Italian children aged 3–12 years with and without autism [33]. Current evidence suggests that humans may lack the gut microbiota with de-epoxidase activity to convert DON to DOM-1 [34] and the accidental transmission of animal microbiota with this capacity to humans may explain the presence of DOM-1 in these populations [15]. 

The present study makes important contributions towards understanding the variation in exposure and possible sources of this variation within an understudied paediatric population. Correlation analysis showed that DON concentrations were higher in children than adolescents. The smaller body size of children and higher relative dietary intake of DON contaminated foodstuff may be possible reasons why children presented with higher DON exposure. These results are also consistent with the inverse correlation between urinary DON concentrations (ng/mg creat) with height and weight, indicating that body size is an important parameter when investigating DON exposure in humans. In contrast, our correlation analysis did not reveal any sex differences in DON concentrations (ng/mg creat) in the urine. These findings agree with some [22,35], but not all previous studies (males > females) [21,36]. Differences in DON metabolism or differences in dietary intake and hence, DON exposure between males and females, have been suggested as possible reasons for sex-specific sensitivity, although current evidence from human studies is limited [37]. 

Another factor that may influence the extent of DON exposure is dietary factors. Wheat and its products such as bread, biscuits, crackers, and pasta represent the main source of DON intake in children’s diet in the EU [38]. Correlation analysis between urinary biomarker levels and food consumption patterns based on 24-h recall data of the days prior to urine sample collections showed significant associations with pizza only and total dietary intake of foodstuff commonly contaminated with DON. These results may have been affected by uncertainties linked to the dietary assessment and analyses method used in the present study. Participants were asked to recall the consumption of food items (frequency) and characterise the quantity of the food consumed as small, medium, and large, with these portion sizes corresponding to predefined grams [20]. Documentation of actual weights consumed could have improved the accuracy of the dietary data recorded and subsequently, our correlation analysis between cereal-based foodstuffs and DON concentrations in urine. Furthermore, in order to better ascertain the influence of cereal foods consumption on the concentration of DON in urine and give strengths to the statistics, an increase of the number of days of recording (four to seven days) is recommended.

## 4. Conclusions

In summary, this study showed moderate mean levels of DON in urine samples of children aged 3–9 years and adolescents aged 10–17 residing in an urban area of the UK. However, the high frequency of DON detection, together with the high maximum biomarker concentrations and great estimated dietary exposure of DON, raise concerns about the high risk of DON exposure in some young individuals within this cohort. Despite the number of uncertainties coming from the percentages of DON excretion rates and urine outputs that still endure in handling biomonitoring data, the study findings underpin the need for larger-scale studies in different areas of the country to provide a comprehensive assessment of the dietary exposure of the British paediatric population to DON and to understand any DON-associated impact on development and health. 

## 5. Materials and Methods 

### 5.1. Participants Selection and Recruitment 

Children aged 3–9 years (*n* = 40) and adolescents aged 10–17 years (*n* = 39) and their parents or guardians residing within Hull and East Yorkshire were recruited via word of mouth, by an approved advertisement in the local papers and a global email circulated through the University of Hull and Hull and East Yorkshire Hospitals NHS portal. Participants were recruited during the period May and October 2014. 

Children aged 3–9 years and adolescents aged 10–17 years were included if they were in good health, were not taking any current medication initiated within the last three months, or were on stable medication (over a duration >3 months). Participants were excluded if they were unable to provide informed consent/complete the questionnaire (participants themselves or their guardians), if they were suffering from acute or chronic illness (chronic renal, hepatic, or cardiac problems, cancer), chronic gastrointestinal conditions (e.g., coeliac disease), gluten sensitivity, eating disorders, depression, psychosis, hospitalisation within three months of enrolment in the trial, or if they were following a weight loss programme. Children and adolescents on stable medication, which may affect appetite such as oral steroids, were also excluded from partaking in the study.

Participants under the age of 16 had their consent forms signed by a parent or guardian, whereas participants over the age of 16 consented for themselves. This study was approved by the National Health Service (NHS), National Research Ethics Service (NRES) Committee Yorkshire & the Humber-Leeds West (IRAS project code: 147707). 

### 5.2. Study Design 

The dataset in the present analysis represents a subset of data collected for a larger study entitled “Experimental study of deoxynivalenol biomarkers in urine” conducted for the European Food Safety Authority (EFSA) GP/EFSA/CONTAM/2013/04 [20]. In brief, this study explored the occurrence of DON and its metabolites in urine from different population groups (children, adolescents, adults, elderly, and pregnant women; total *n* = 635) in three European countries (UK, Italy, and Norway) and the relationships between urinary DON levels and its metabolites and self-reported dietary intake of cereal-based food items. 

Demographic (age, sex), anthropometric (height, weight) together with habitual dietary and physical activity data were recorded as part of the food frequency questionnaire (FFQ) utilised in [20]. For a detailed assessment of the main dietary sources of DON exposure, each participant completed a food record over a 24-h period on the days prior to the collection of their first morning urine sample. Each 24-h food record consisted of meal sections for the following eating occasions; breakfast, lunch, dinner, and snacks. Each meal section included examples of food items which belonged to seven food categories, all known to contribute to the consumption of DON. These food categories were in accordance with the validated FFQ used to assess the main dietary sources of DON [18,19,20] and included breakfast cereals and snacks, bread, products alternative to bread, pasta, biscuits and bakery products, cereals and similar, and beer. For consistency and ease of recording, the example food items within each category were tabulated with tick boxes as to whether the portion consumed was small, medium, or large. To further enhance participants’ accuracy to quantify the food items they consumed, photographic examples of portion sizes were used. 

Urine samples were collected on two consecutive days from each participant in order to explore potential between-day variability and increase the reliability and accuracy of our results. Collection containers (four urine sample pots of 50 mL) and written instructions were given all participants, who provided a sample of first morning urine, in their own home, on two consecutive days. The urine samples were returned on the same day and centrifuged at the Hull Royal Infirmary (Hull, UK) immediately, in a refrigerated centrifuge at 2000 rpm for 10 min. The samples were stored at −80 °C until they were further analysed for DON and its metabolites by using the HPLC-MS/MS methodology, developed and validated at Leeds University [12]. 

### 5.3. Laboratory Analysis

The following chemicals were used for the analysis of DON and its metabolites: ^13^C labelled DON standard (Sigma, Saint Louis, MO, USA; product number: 34128, 1.2 mL); DON (Sigma, Saint Louis, MO, USA; product number: D0156, 1 mg); β-glucuronidase (Type IX-A from *E. coli*; Sigma, Saint Louis, MO, USA; product number: G7396-2MU); DOM-1 (Sigma, Saint Louis, MO, USA; product number: 34135, 2 mL); and DONtest WB^TM^ immunoaffinity columns (Vicam, Milford, MA, USA; product number: G1066). The equipment used was a Waters 2795 HPC Separation Module (Waters Corp., Milford, MA, USA) with a Quattro Micro Triple Quadrupole Mass Spectrometer (Micromass UK Ltd., Manchester, UK).

#### 5.3.1. Sample Preparation

Urinary DON and metabolites concentrations were measured using a two-step process. Stored urine samples were allowed to thaw, and were then centrifuged (2000 rpm; −4 °C; 15 min). For each participant, two aliquots (1 mL) were prepared by mixing ^13^C-DON internal standard solution, to provide a final concentration of 20 ng/mL. Aliquot 1 was used to determine total DON concentrations, defined as the sum of glucuronide metabolites and free DON. To measure combined glucuronide metabolites of DON (DON-3-GlcA and DON-15-GlcA) and free DON, each sample was adjusted to pH 6.8 and digested using β-glucuronidase solution (23,000 units, in KH2PO4 75 mM) in a shaking water bath at 37 °C for 18 h. After this period, the samples were removed, centrifuged (2000 rpm; −4 °C; 15 min), and the supernatant was diluted to a final 4 mL with phosphate buffered saline (PBS, pH 7.4). The diluted urine sample was passed through a wide bore DON immunoaffinity column. DON was eluted from columns with methanol (4 mL) and extracts were dried under vacuum using a SavantTM SpeedVacTM (Thermo Fisher Scientific Inc., Waltham, MA, USA) or equivalent and reconstituted in 10% ethanol (250 μL) for LC-MS analysis. DOM-1 was quantified on the same aliquot analysed for DON-GlcA. Aliquot 2 was used to assess free DON using the aforementioned procedures, but without any β-glucuronidase treatment.

#### 5.3.2. HPLC-MS Analysis: DON Determination

The separation of DON was performed by utilising reversed phase chromatography conditions using a Luna C_18_ column (150 × 4.6 mm, 5-μm particle size (Phenomonex, Macclesfield, UK) with a mobile phase sequence of 27 min 20% methanol, changing to a wash of 75% methanol after 10 min, and followed by 20% methanol after 16 min (flow rate 1 mL/min; injection volume 25 μL). One fifth of the eluent was placed into the desolvation chamber of the MS and the rest was withdrawn as waste. The quantification of DON by reference to ^13^C-DON internal standard was performed using selective ion recording (SIR). The following mass spectrometer conditions were kept constant: capillary voltage 3.5 kV, desolvation temperature 300 °C, extraction cone voltage 3.00 V, sampling cone voltage 35.00 V, source temperature 100 °C, cone gas flow 50 L/h, collision energy 1.0, and desolvation gas flow 500 L/h. Two masses of DON ([DON-H]+, *m*/*z* 297.2 and [DON-Na]+, *m*/*z* 319.2) and ^13^C-DON ([^13^C-DON-H]+, *m*/*z* 312.2 and [^13^C-DON-Na]+, *m*/*z* 334.2) were monitored for 0.25 s (each mass) and they were subsequently summed to create a total ion current peak for the internal standard and each analyte. The calibration curve was set by the injection of DON and ^13^C-DON standard solution (prepared in 10% ethanol) and covered the range between 2 ng/mL and 250 ng/mL.

DON-GlcA values were estimated indirectly, by subtracting free DON values from total DON values for each participant.

#### 5.3.3. LC-MS Analysis: DOM-1 Determination

To separate DOM-1, the same chromatographic column utilized for DON separation was used together with a mobile phase sequence of 35 min 20% methanol, changing to a wash of 75% methanol after 20 min, and returning to 20% methanol after 26 min (injection volume 25 L; flow rate 1 mL/min). One fifth of the eluent was driven into the desolvation chamber of the MS. SIR was performed to quantify DOM-1 by reference to the calibration curve, obtained by the injection of DOM-1 standard solutions (prepared in 10% ethanol) within the range of 2–200 ng/mL, using the least squares regression analysis. Two masses of DOM-1 ([DOM-1-H]+, *m*/*z* 281.3 and [DOM-1-Na]+, *m*/*z* 303.3) were monitored for 0.25 s (each mass) and summed to attain a total ion current peak for DOM-1. The capacity and efficiency of the chromatographic column used in this study to retain DOM-1 has been previously assessed [15] and shown to capture 80–85% of DOM-1, thus, slightly underestimating DOM-1 concentrations. In the present analysis, this is, however, unlikely, given that no signal was detected.

#### 5.3.4. Creatinine Analysis

DON concentrations were adjusted for creatinine to correct for variable dilutions between individuals, given that only first morning samples of urine were collected. Urinary creatinine analysis was conducted using an in-house micro-titre plate assay [39]. Urine samples were diluted in water (1:20) and 100 µL was loaded into a 96-well plate, in duplicate. A duplicate standard curve of creatinine concentrations was also prepared per plate (range between 0 and 20 μg/mL). Then, 100 μL of alkaline picric acid solution was added to each well, incubated at 25 °C for 30 min, and read at 490 nm using a plate spectrophotometer. Total DON concentrations in urine samples are presented as unadjusted (ng/mL) and adjusted for creatinine (ng/mg creatinine). 

### 5.4. Estimated Dietary Intake of DON Based on Urinary Levels and Comparison with PMTDI 

The dietary intake of DON was estimated using the following formula [21,28]:

Estimated dietary intake DON (μg/kg b.w./day) = $\frac{\mathrm{total}\;\mathrm{DON}\times V}{\mathrm{ER}\times b.w.}\times 1000$
ER = urinary excretion rate of DON is 72% [13].
V = estimated urine volume (mL/day) for children and adolescents. Minimum, maximum, and mean values within the range provided for children (1–2 mL/kg b.w./h) and adolescents (0.5–1 mL/kg b.w./h) [25] were tested.
b.w. = body weight (kg) reported in the questionnaire

### 5.5. Dietary Analysis

Portion sizes recorded in the food diaries were translated into grams by research dietitians before the data were entered into the FoodEx2 coding system [40,41]. FoodEx2 (revision 1) is a detailed system used for food classification and description across several food safety domains directed by EFSA. In brief, FoodEx2 contains a main food list or generic descriptions of food items as a minimum level of detail required for intake or exposure assessments [42].

### 5.6. Statistical Analysis 

The Shapiro–Wilk test was used to check the distribution of the quantitative data. Differences in urinary DON levels between day 1 and day 2 for children aged 3–9 years and adolescents aged 10–17 years were analysed using a paired *t*-test for normally distributed data and Wilcoxon signed-rank test data for non-parametric data. Differences in urinary concentrations of DON and its metabolites between children aged 3–9 years and adolescents aged 10–17 years were analysed by an independent sample *t*-test or Mann-Whitney two sample statistics for parametric and non-parametric data, respectively. The Spearman correlation coefficient (two-tailed) was used to assess the correlation between urinary DON levels and its metabolites and demographic (i.e., sex), anthropometric (i.e., weight, BMI), and dietary factors of the participants. All analyses were carried out using IBM SPSS Statistics Version 23.0. Findings were considered significant at *p*-value < 0.05.

## Figures and Tables

**Table 1:** Non-adjusted and creatinine-adjusted total DON concentrations in urine samples by day and sex in children aged 3–9 years and adolescents 10–17 years.

Age Group	Day	Gender ^(a)^	Total DON (ng/mL Urine)			Total DON (ng/mg Creatinine)		
			Mean	Range ^(b)^	P50–P75	Mean	Range ^(b)^	P50–P75
Children (3–9 years) (*n* = 40)	1	F (20)	38.2	3.0–138	22.5–47.1	56.3	6.8–214.0	44.1–73.9
		M (20)	22.5	1.2–86.5	18.3–31.0	33.9	5.3–130.0	25.3–37.3
	2	F (20)	35.4	1.4–140.9	23.7–51.0	43.8	8.0–219.0	23.1–52.9
		M (20)	20.6	4.1–52.0	15.9–32.4	32.1	8.2–88.4	20.6–44.3
Adolescents (10–17 years) (*n* = 39)	1	F (20)	26.0	3.1–104.3	19.6–30.8	19.4	2.1–58.0	15.4–27.3
		M (19)	20.6	1.6–52.5	18.2–30.9	19.0	1.8–45.3	15.0–31.9
	2	F (20)	28.8	0–67.2	24.9–48.0	20.8	0–51.8	18.5–25.1
		M (19)	27.0	2.9–66.3	19.7–40.2	25.0	2.2–95.6	14.5–32.9

Data are presented as mean, range (minimum–maximum), and 50th (median) to 75th percentile values (P50–P75). DON: deoxynivalenol; min: minimum; max: maximum; P50: 50th percentile; P75: 75th percentile. ^(a)^ F, Female; M, Male; In parenthesis, number of subjects. ^(b)^ Range (minimum to maximum).

**Table 2:** Creatinine-adjusted Free DON, DON-GlcA, and DOM-1 levels in urine samples by day and sex in children and adolescents in the UK.

Age Group	Day	Gender ^(a)^	Free DON (ng /mg Creatinine)	DON-GlcA (ng /mg Creatinine)	DOM-1 (ng /mg Creatinine)
Children (3–9 years) (*n* = 40)	1	F (20)	12.4 (0.0–51.0)	43.9 (5.0–168)	nd
		M (20)	7.5 (1.5–32.8)	26.5 (3.8–97.5)	nd
	2	F (20)	6.8 (0.0–55.2)	38.2 (6.0–182)	nd
		M (20)	6.8 (1.1–29.4)	25.2 (7.1–59.8)	nd
Adolescent (10–17 years) (*n* = 39)	1	F (20)	2.8 (0.0–10.2)	16.7 (2.1–47.9)	nd
		M (19)	3.7 (0.0–8.9)	17.6 (0.0–45.2)	nd
	2	F (20)	2.7 (0.0–6.9)	15.3 (1.8–37.7)	nd
		M (19)	5.3 (0.0–17.0)	19.7 (1.1–78.6)	nd

Data are presented as mean (range: minimum–maximum). DON: deoxynivalenol; DON-GlcA: deoxynivalenol glucuronide; DOM-1: deepoxy-deoxynivalenol; min: minimum; max: maximum; nd refers to levels below the limit of quantification (LOQ). LOQ for free DON was 0.25 ng/mL and for DON-GlcA and DOM-1 0.50 ng/mL. ^(a)^ F, Female; M, Male; In parenthesis, number of subjects.

**Table 3:** Estimated dietary intake of DON based on urinary levels. The intake was compared to the PMTDI.

Age Group	Estimated Urine Volume	DON (μg/kg·b.w./Day)	% Exceeding TDI
Children (3–9 years) (*n* = 40)	1 mL/kg b.w./h	0.97 (0.14–4.6)	33 (13/40)
	1.5 mL/kg b.w./h	1.46 (0.20–6.96)	50 (20/40)
	2 mL/kg b.w./h	1.95 (0.30–9.28)	63 (25/40)
Adolescents (10–17 years) (*n* = 39)	0.5 mL/kg b.w./h	0.47 (0.03–1.22)	5 (2/39)
	0.75 mL/kg b.w./h	0.64 (0.04–1.75)	21 (8/39)
	1 mL/kg b.w./h	0.95 (0.05–2.44)	46 (18/39)

Data are presented as mean (range: minimum–maximum). Estimated urine volume as reported in [25], minimum, maximum, and mean values of the proposed range were used. PMTDI: Provisional Maximum Tolerable Dietary Intake of 1 μg/kg b.w./day [6].

**Table 4:** Consumption of foods that commonly contribute to dietary DON exposure.

Food Category	Children (3–9 Years) (*n* = 40)	Adolescents (10–17 Years) (*n* = 39)
Bread (g/d) ^(a)^	107 ± 62 (23–315)	95 ± 58 (0–225)
Breakfast cereals (g/d)	20 ± 15 (0–65)	19 ± 16 (0–63)
Sweet snacks (g/d) ^(b)^	44 ± 33 (0–112)	57 ± 55 (0–198)
Pizza (g/d)	19 ± 28 (0–85)	31 ± 53 (0–213)
Pasta (g/d)	63 ± 62 (0–190)	62 ± 76 (0–290)
Total (g/d)	252 ± 89 (95–454)	264 ± 102 (116–516)

Data are presented as mean ± 1SD (range: minimum-maximum). ^(a)^ Wheat bread and rolls (white with refined flour, white with maize, brown, or wholemeal), flatbreads, and pita bread; ^(b)^ Biscuits, pancakes, and baked fine wares.
